# Updated Australian diagnostic reference levels for adult CT

**DOI:** 10.1002/jmrs.372

**Published:** 2020-02-10

**Authors:** Kam L. Lee, Toby Beveridge, Masoumeh Sanagou, Peter Thomas

**Affiliations:** ^1^ Australian Radiation Protection and Nuclear Safety Agency Yallambie Victoria Australia

**Keywords:** Computed tomography, diagnostic reference level, dose survey, optimisation, radiation protection

## Abstract

**Introduction:**

In 2018, ARPANSA published updated national DRLs for adult CT, which were first published in 2012, and augmented the national DRL categories. This paper presents the updated national DRLs and describes the process by which they were produced.

**Methods:**

Examine patient survey data submitted to the Australian Radiation Protection and Nuclear Safety Agency (ARPANSA) National Diagnostic Reference Level Service (NDRLS). Determine the quartiles of the distributions of median survey dose metrics with categorisation by procedure type. Engage a liaison panel representing the radiology professions to review procedure categories and recommend revised national DRLs. The revised NDRL procedure categories are: head (non‐contrast brain (trauma/headache)), cervical spine (Non‐contrast (trauma)), soft‐tissue neck (post‐contrast (oncology)), chest (post‐contrast (oncology)), abdomen–pelvis (post‐contrast (oncology)), kidney–ureter–bladder (non‐contrast (suspected renal colic)), chest–abdomen–pelvis (post‐contrast (oncology)) and lumbar spine (non‐contrast (degenerative pain)).

**Results:**

The existing six procedure categories were revised and refined. Updated Australian national diagnostic reference levels for adult CT were recommended and endorsed for eight procedure categories: head (52 mGy/880 mGycm), cervical spine (23 mGy/470 mGycm),soft‐tissue neck (17 mGy/450 mGycm), chest (10 mGy/390 mGycm), abdomen–pelvis (13 mGy/600 mGycm), kidney–ureter–bladder (13 mGy/600 mGycm), chest–abdomen–pelvis (11 mGy/940 mGycm) and lumbar spine (26 mGy/670 mGycm). The updated national DRLs are between 12 and 26% lower than the previous DRLs for dose‐length product and between 13 and 63% lower for volume computed tomography dose index.

**Conclusions:**

Australian national DRLs for adult CT have been reviewed and revised. The revised national DRLs are lower, better reflecting current practice among imaging facilities in Australia. The revised Australian national DRLs are similar to those in other developed countries.

## Graphical abstract

Australian national DRLs for adult CT have been reviewed and revised. Revised DRLs are set at the 75th percentile of the distribution of facility medians in 2017 data. The revised DRLs better reflect current practice and are similar to those in other developed countries.

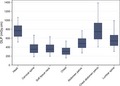

## Introduction

The use of Multi‐Detector Computed Tomography (MDCT) in diagnosis and therapy is indisputably of great benefit, however its application is presumed to carry some risk of cancer and other effects due to radiation exposure.^1^ Accordingly, MDCT scan providers must optimise acquisition protocols to ensure that radiation exposure is as low as reasonably achievable yet sufficient to deliver the desired imaging result.

To identify facilities that could benefit from optimisation of acquisition protocols, it is considered international best practice for regulatory and health authorities, in partnership with relevant professional associations, to promulgate Diagnostic Reference Levels (DRLs).

DRLs are a type of investigation level used in medical imaging to indicate whether, in routine conditions, the amount of radiation used for a type of procedure is unusually high or low. DRLs are based on dosimetry surveys that measure the spread of doses used for similar radiological investigations across various institutions.^2^, ^3^, ^4^, ^5^, ^6^, ^7^ Individual sites then compare, on a regular basis, typical values of the relevant dose indices at their site with the DRLs. Doses for individual patients are not compared with the DRLs, rather the typical value (usually mean or median) from a representative sample of patients is compared. If a site’s typical dose index value exceeds the corresponding DRL, this is an indication that additional optimisation may be required.

The establishment of DRLs has proved to be a useful tool in the standardisation and optimisation of radiation doses received from common medical imaging protocols.^8^, ^9^, ^10^, ^11^, ^12^, ^13^ In order for DRLs to remain relevant, they need to be periodically updated to reflect changes in practice within the given jurisdiction. In the United Kingdom, national surveys of radiographic facilities have been conducted every five years since the mid‐1980s.^14^ Improved optimisation has resulted in an overall lowering of doses with each iteration of the dose review.^8^, ^15^

In Australia, the Australian Radiation Protection and Nuclear Safety Agency (ARPANSA) is responsible for setting National DRLs (NDRLs) and conducts an ongoing national dosimetry survey of common MDCT protocols for this purpose.^16^, ^17^

Since the first publication of the Australian NDRLs for adult MDCT in 2012,^18^ MDCT technologies have changed significantly and together with dose optimisation initiatives, this necessitated a review of the Australian NDRLs. In addition, the International Commission on Radiological Protection (ICRP) recommends that NDRLs should be reviewed at regular intervals (3–5 years).^19^ The NDRLs were updated for the first time in July 2018. This paper presents the method and results of this review together with the revised Australian NDRLs.

## Methods and Materials

ARPANSA’s National Diagnostic Reference Level Service (NDRLS) provides an online survey for registered facilities to submit patient dosimetry information. The online service collects volume computed tomography dose index (CTDI_vol_), dose length product (DLP), patient weight and gender, scanner information and protocol information (e.g. contrast, number of phases, technique factors, reconstruction method and dose modulation information). The details of the service have been described in a previous publication.^18^ As a quality improvement activity, this project was exempt from research ethics approval.

A given survey contains dose metrics for a sample of up to 20 patients for a particular protocol conducted on a particular scanner at a particular site. The median values of the dose metrics in a survey are reported as the typical values for that combination of protocol, scanner and site. ARPANSA uses the term Facility Reference Levels (FRLs) to refer to these site‐scanner‐protocol‐specific survey medians. In Australia, the NDRLs have been set at rounded third quartiles (75th percentiles) of the distributions of FRLs, the implication being that sites with an FRL above the DRL deliver a dose greater than that of 75% of their peers. This is consistent with ICRP recommendations^19^ and addresses variations across facilities at a national level.

Surveys can be initiated at any time and remain open to accumulate patient data progressively before manual submission at a later date. At the end of a given calendar year all remaining open surveys are automatically closed and a facility must create new surveys for the following calendar year. In return for submitting data to the NDRLS, participants receive a report comparing their FRL with the relevant DRL, which can in turn be used to demonstrate compliance with state regulations or Diagnostic Imaging Accreditation Scheme (DIAS) requirements.^20^

ARPANSA began a review of the data collected by the NDRLS at the end of 2017. In analysing the FRL distributions to derive DRLs, each survey median (FRL) was treated as an independent sample. Analysis was restricted to surveys where the median patient age was >20 years and to single‐series studies, except in the case of chest–abdomen–pelvis studies where both single‐series and dual‐series scans were included. Surveys with a median age of less than 20 were excluded to remove a small number of submissions from paediatric facilities in which all patients were aged between 15 and 18 years because these represent a potentially distinct patient population in comparison with the general adult population.

The results of the review were provided to a liaison panel who were tasked with advising on the new Australian DRLs. In addition to setting new NDRL values, the panel was given the scope to alter the NDRL categories and definitions. The panel comprised representatives from ARPANSA, the Australian Government Department of Health (DoH), the Royal Australian and New Zealand College of Radiologists (RANZCR), the Australasian College of Physical Scientists and Engineers in Medicine (ACPSEM), the Australian Diagnostic Imaging Association (ADIA) and the Australian Society of Medical Imaging and Radiation Therapy (ASMIRT). The liaison panel reached their recommendations by consensus with subsequent formal endorsement by all participating bodies.

The use of a liaison panel of industry experts to guide the DRL program ensured that the development of DRLs was a collaborative process. A similar panel was used to develop the initial MDCT NDRLs.^18^ Cooperation between regulatory authorities and professional groups in establishing DRLs is consistent with the requirements of the IAEA International Basic Safety Standards.^21^

The liaison panel considered setting separate NDRLs with and without the use of iterative reconstruction but favoured the simplicity of keeping a single NDRL for each protocol, deciding to continue with categorisation at the protocol level. The revised NDRLs were chosen by rounding the third quartiles of the 2017 FRL distributions up to the nearest integer for CTDI_vol_ and to the nearest factor of 10 for DLP. In addition, the liaison panel requested that the existing neck protocol be split into separate cervical spine and soft tissue neck protocols and that a new category be introduced for kidney–ureter–bladder (KUB) scans for the assessment of renal colic. These alterations were made to better reflect clinical practice and to reduce ambiguity in scan definition.

**Figure 1:**
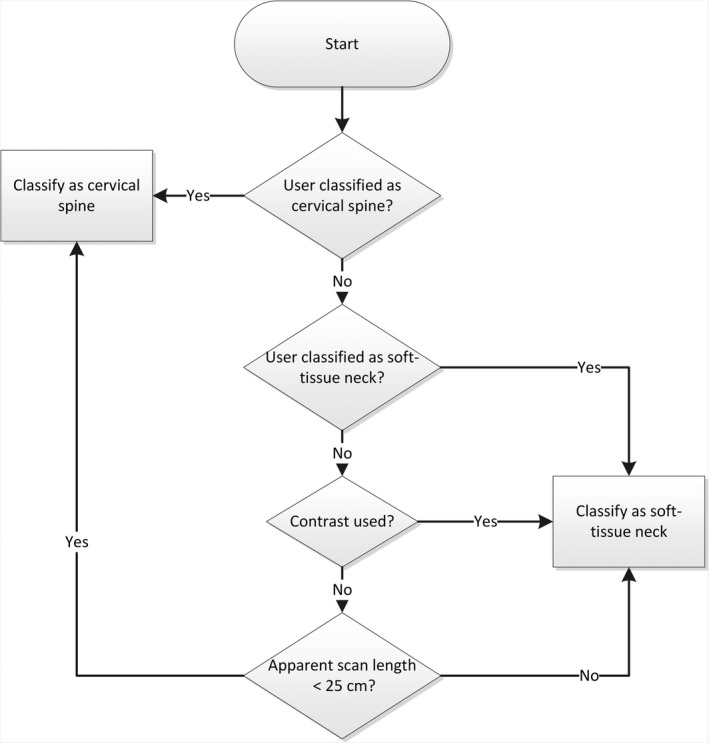
Process for reclassifying the neck category into cervical spine and soft‐tissue neck.

Since the NDRLS only collected data for a unified ‘neck’ protocol, it was necessary to re‐categorise the existing neck surveys into the new cervical spine and soft‐tissue neck categories. Classification was based on: supplementary information about the procedure supplied by the facility in the NDRLS survey comment field, the use of contrast and the apparent scan length. The apparent scan length was obtained by dividing the DLP by the CTDI_vol_. Figure 1 depicts the flow of this classification process.

For KUB scans, ARPANSA did not have access to any relevant dose data. Instead, a provisional DRL was set using the same values as were used for abdomen–pelvis scans.

For the chest–abdomen–pelvis (CAP) category, it was thought that a number of survey participants had submitted incorrect information. The issue arose from facilities submitting data from scans that required multiple series to cover the entire CAP scan length. In these instances, rather than submit the sum of the DLP and the average CTDI_vol_ across all series as requested in the NDRLS user instructions, many participants submitted the sum of the CTDI_vol_. Data following this pattern resulted in the 2012 CAP CTDI_vol_ DRL being double the abdomen–pelvis and chest CTDI_vol_ values. To correct for this error, the reported CTDI_vol_ was halved for any surveys with a median apparent scan length <55 cm.

Nonparametric quantile regression models were used to test the differences between the third quartiles of FRLs for data submitted to the ARPANSA NDRLS in 2011 and 2017. Quantile regression is a method that makes no assumptions about the underlying data distribution. A two‐tailed p‐value of 0.05 was used to indicate statistical significance. Statistical analyses were performed using Stata/SE V.16.0 (StataCorp LLC, College Station, TX, USA).

## Results

The number of surveys completed for each protocol for adult patients in each year and the proportion of such surveys that indicated the use of iterative reconstruction (IR) is outlined in Table 1. The field indicating the use of IR was added to the NDRLS in April 2013.

**Table 1:** Number of patient dose surveys and proportion using iterative reconstruction (IR) for adult patients submitted to the ARPANSA National Diagnostic Reference Level Service by procedure category and year.

Scan region	2011	2012	2013	2014	2015	2016	2017	Total
Head	56	113	166	147	202	284	465	1433
Neck	30	57	80	76	141	192	358	934
Chest	44	78	112	113	177	258	422	1204
Abdomen pelvis	51	100	150	128	194	274	442	1339
Chest abdomen pelvis (CAP)	40	68	100	93	135	200	368	1004
Lumbar spine	34	75	116	105	156	237	419	1142
Total	255	491	724	662	1005	1445	2474	7056
Proportion using IR	*	*	0.71*	0.65	0.70	0.76	0.84	

^*^ A field to identify the use of iterative reconstruction was added in April 2013.

**Figure 2:**
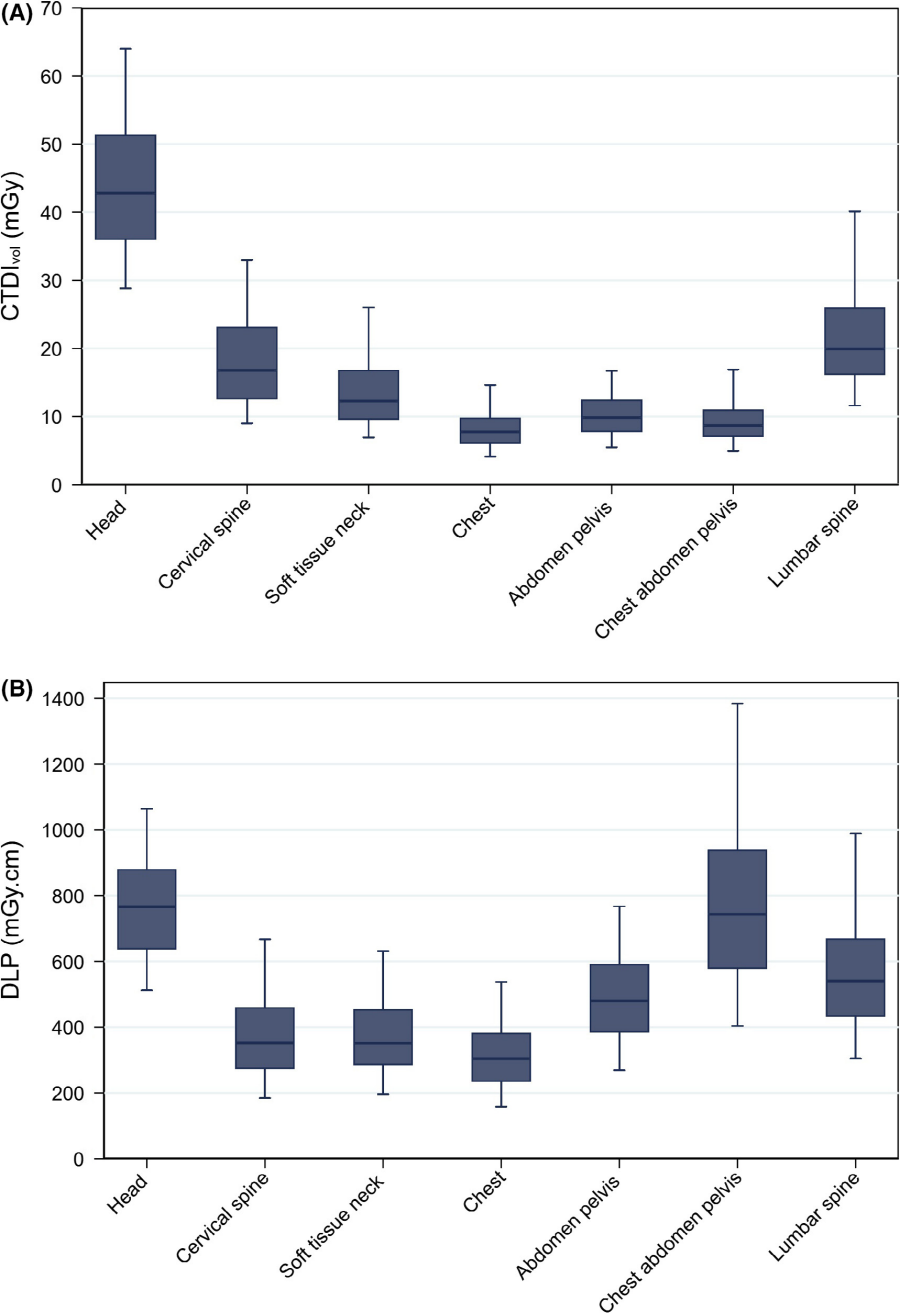
Box plot representations of the (A) CTDI_vol_ and (B) DLP distributions of the data submitted to the NDRLS during 2017, classified by scan region. The whiskers represent the 5th and 95th percentiles of the distributions, and the boxes show the 25th–75th percentile range with a line at the median.

The distributions of FRLs in 2017 for each dose metric, categorised by protocol, are shown in Figure 2. The box plots display the median and interquartile range, with the whiskers indicating the 5^th^ and 95^th^ percentiles.

**Figure 3:**
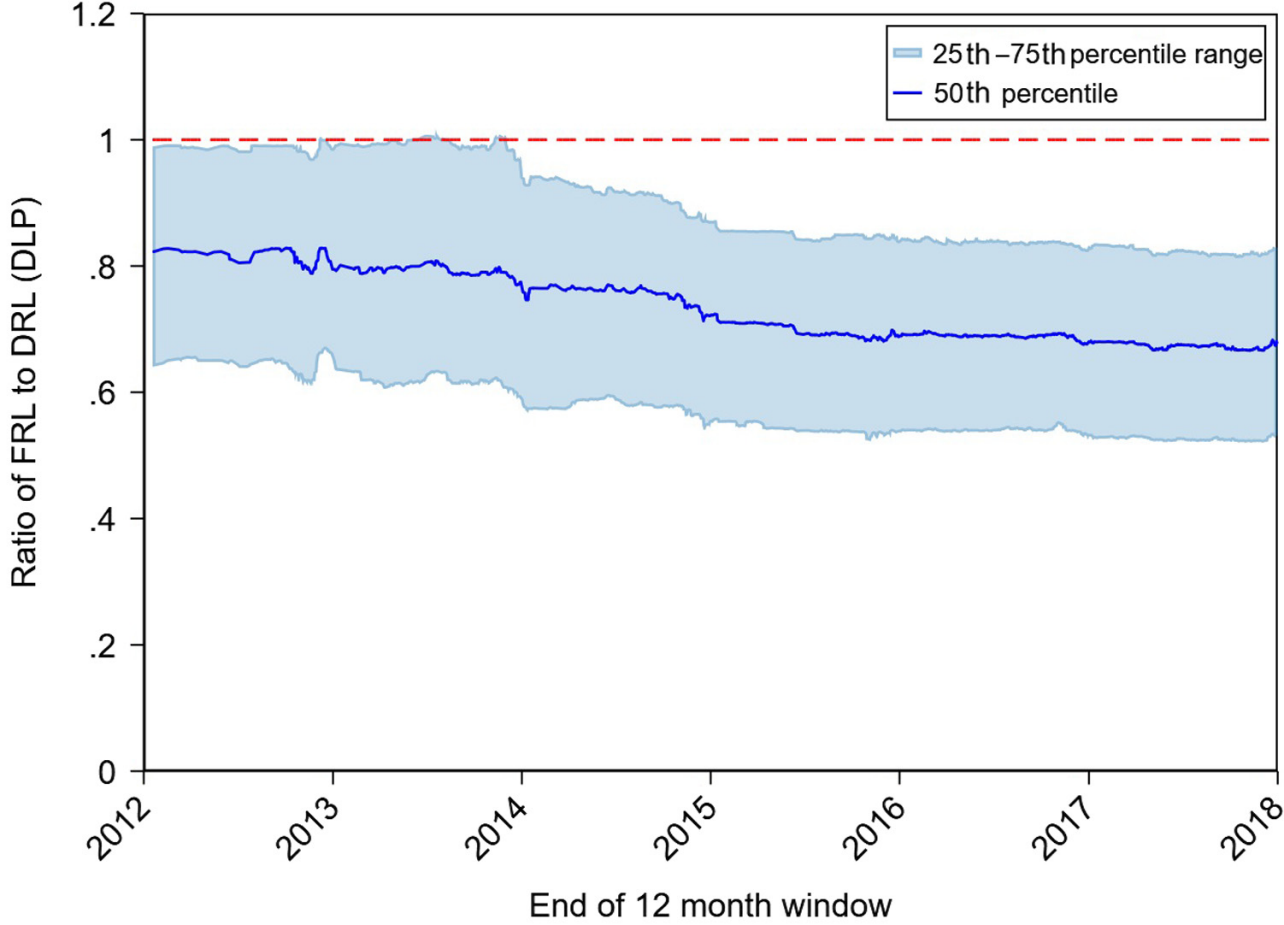
The ratio of FRL to DRL for all scan regions from the beginning of 2012 to the end of 2017. The figure shows a rolling 12‐month median (from the 12 months ending at the date on the *x*‐axis) of the FRL distribution as a solid line with the corresponding interquartile range indicated by the shaded region. The dashed red line indicates the NDRL published in 2012.

The historical variation of the FRL distribution for all protocols is shown in Figure 3 using normalised data. Each FRL is normalised to the NDRL of the corresponding category (using the 2012 NDRLs) and the distribution is determined over all adult surveys submitted in the 12‐month period prior to the given date on the x‐axis. Plots of the historical variation for each individual protocol and dose metric are available on the ARPANSA website.^22^

**Figure 4:**
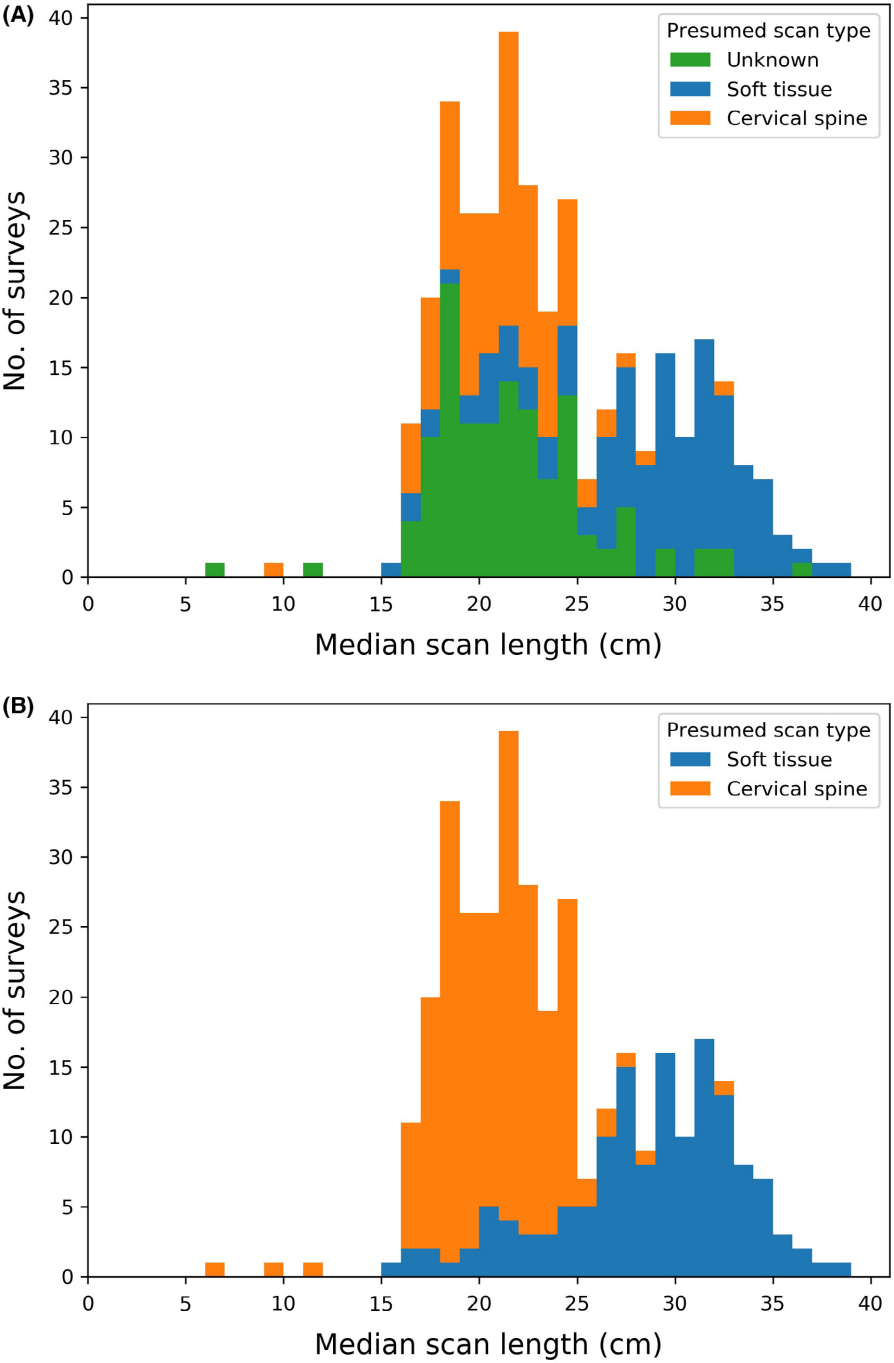
Stacked histograms of the median apparent scan lengths of neck surveys submitted during 2017 to the NDRLS MDCT survey. The scans have been split by presumed clinical task (cervical‐spine vs. soft‐tissue) using (A) the user comments and the use of contrast agent and (B) also the apparent scan length.

Figure 4 (A) shows a stacked histogram of the apparent scan length for all neck scans, categorised by the presumed scan type based on information in the survey comment field or the use of contrast agent. The remaining ‘unknown’ scans were assumed to be soft‐tissue if the apparent scan length was above 25 cm and cervical‐spine if the apparent scan length was below 25 cm, resulting in the distribution in Figure 4 (B).

**Figure 5:**
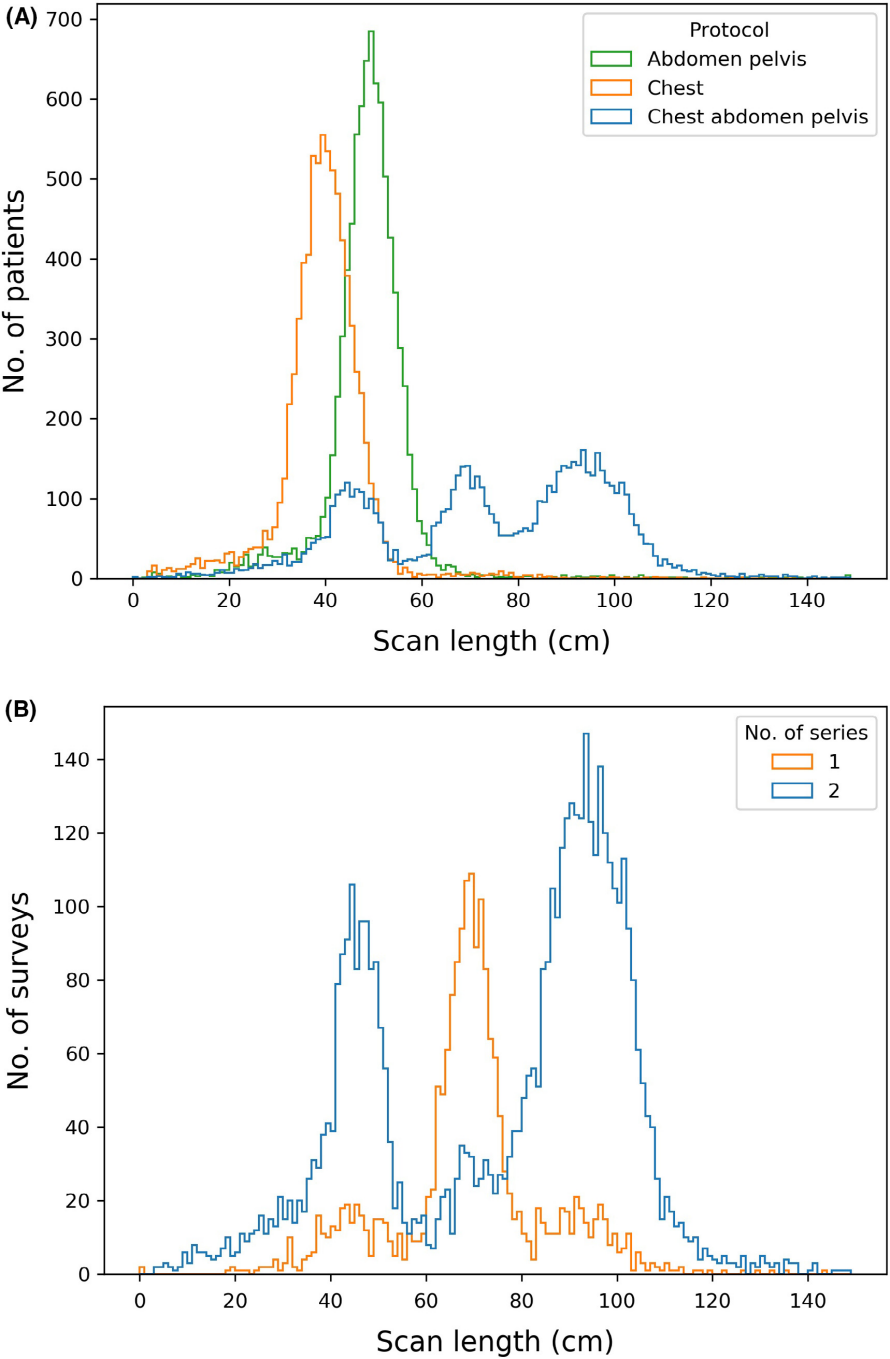
(A) Histogram of apparent scan lengths for three protocols and (B) the CAP apparent scan length distribution separated by number of series.

Figure 5 (A) demonstrates that there is a trimodal distribution for the apparent scan length of CAP scans, and that one of the CAP peaks is centred below the median length of an abdomen–pelvis scan. Figure 5 (B) separates the CAP scans into single and dual‐series scans.

The updated Australian NDRLs are displayed in Table 2. The 25th and 50th percentiles of the FRL distribution, the updated NDRLs and the previous NDRLs (where applicable) are shown in Table 3. All protocols had significant reductions in the third quartiles of their FRLs in 2017 compared to 2011 (Table 4). Table 5 compares the updated Australian NDRLs with those in other jurisdictions.

**Table 2:** New Australian NDRLs for adult CT.

Scan region	Description	CTDI_vol_ (mGy)	DLP (Gy⋅cm)
Head	Non‐contrast brain (trauma or headache)	52	880
Cervical spine	Non‐contrast (trauma)	23	470
Soft‐tissue neck	Post‐contrast (oncology)	17	450
Chest	Post‐contrast (oncology)	10	390
Abdomen–pelvis	Post‐contrast (oncology)	13	600
KUB	Non‐contrast (suspected renal colic)	13	600
CAP	Post‐contrast (oncology)	11	940
Lumbar spine	Non‐contrast (degenerative pain)	26	670

CAP, chest–abdomen–pelvis; KUB, kidney–ureter–bladder.

**Table 3:** FRL distribution percentiles and comparison with the previous NDRLs.

Scan region	CTDI_vol_ (mGy)					DLP (mGy⋅cm)				
	25th	50th	New NDRL	2012 NDRL	Difference (%)	25th	50th	New NDRL	2012 NDRL	Difference (%)
Head	36	43	52	60	−13.3	640	770	880	1000	−12.0
Cervical spine	13	17	23	(30)^1^		270	350	470	(600)^1^	
Soft‐tissue neck	9.5	12	17	(30)^1^		290	350	450	(600)^1^	
Chest	6.0	7.7	10	15	−33.3	230	300	390	450	−13.3
Abdomen–pelvis	7.7	9.8	13	15	−13.3	380	480	600	700	−14.3
KUB			13					600		
CAP	7.0	8.7	11	30	−63.3	580	740	940	1200	−21.7
Lumbar spine	16	20	26	40	−35.0	430	540	670	900	−25.6

CAP, chest–abdomen–pelvis; KUB, kidney–ureter–bladder.

New NDRL is the 75th percentile of the FRLs.

^1^ Previously there was only a single category for neck which has now been split into cervical spine and soft‐tissue neck.

**Table 4:** Quantile regression results for comparisons of the 75th percentile of the distributions of FRLs in 2011 and 2017.

Scan region	Difference in the third quartiles of the distributions of FRLs in 2011 and 2017			
	DLP	*P*‐value	CTDI_vol_	*P*‐value
Abdomen Pelvis	−105	0.007	−4.8	<0.001
Chest	−79	0.014	−4.7	<0.001
Chest Abdomen Pelvis	−194	0.001	−4.9	<0.001
Head	−91	0.014	−9.5	0.001
Lumbar Spine	−216	0.001	−17.3	<0.001
Soft Tissue Neck	−200	0.015	−9.8	0.017
Cervical Spine	−121	0.032	−12.0	0.003

**Table 5:** Comparison of the new Australian NDRLs with those of other countries.

Scan region	Australia		UK^23^		USA^24^		Germany^15^		Korea^25^		Japan^26^	
	CTDI_vol_	DLP	CTDI_vol_	DLP	CTDI_vol_	DLP	CTDI_vol_	DLP	CTDI_vol_	DLP	CTDI_vol_	DLP
	(mGy)	(mGy⋅cm)	(mGy)	(mGy⋅cm)	(mGy)	(mGy⋅cm)	(mGy)	(mGy⋅cm)	(mGy)	(mGy⋅cm)	(mGy)	(mGy⋅cm)
Head	52	880	60	970	56	962	60	850	63	1119	85	1350
Cervical spine	23	470	28	600	28	562	20	300	18	434		
Soft‐tissue neck	17	450			19	563	15	330	14	442		
Chest	10	390	12	610	13	469	10	350	7	297	15	550
Abdomen–pelvis	13	600	15	745	15	755	15	700	10	472	20	1000
KUB	13	600	10	460								
CAP	11	940		1000	15	947	13	1000			18	1300
Lumbar spine	26	670					10	180	18	601		

CAP, chest–abdomen–pelvis; KUB, kidney–ureter–bladder.

## Discussion

The number of surveys submitted to the NDRLS in each protocol category has increased from around 40–50 in 2011 to around 400 in 2017 (Table 1). A recent Australian Senate inquiry into the availability and accessibility of diagnostic imaging showed a total of 1507 CT scanners across the country, although this may include scanners used for purposes other than routine diagnostic imaging, such as radiotherapy treatment planning.^27^ On this basis, the NDRLS data has increased from a sample of about 3% of all scanners to around 25–30%. The growth in survey contributions is an encouraging sign of engagement with the DRL program and gives greater confidence that the data on which the NDRLs are based are representative. It should be noted, however, that data submission to the ARPANSA NDRLS is not compulsory and the results could therefore be biased.

The observed FRL distributions have evolved over time (Figure 3). The third quartile remained close to the initial NDRL for around 2 years, then began to decline in 2014 and 2015, and has since stabilised at the current level, which is reflected in the new NDRLs. The median and the first quartile of the distribution have shown a similar evolution over the same period.

The use of IR has become increasingly prevalent in the survey data submitted to the NDRLS (Table 1). Tables of the quartiles of the FRL distributions categorised by the use of IR or filtered back projection are available on the ARPANSA website.^22^ ARPANSA recommends that facilities use these tables to make a more nuanced comparison of their data with the FRL distribution from sites using the reconstruction technique that more closely matches their own.

The neck scan region defined in the 2012 NDRLs covered scans of the cervical spine and also soft‐tissue scans. Figure 4 shows that both types of scans were included in ‘neck’ data submitted to the NDRLS. The distribution is bimodal with a peak at around 20 cm and a second peak at around 30 cm. As expected, those scans that are presumed to be soft tissue are generally longer than those presumed to be of the cervical‐spine. There will be cases where surveys have been misclassified; however, ARPANSA believes the overall CTDI_vol_ and DLP distributions should still be representative.

Similar to the data collected in the USA by the American College of Radiology’s (ACR) Dose Index Registry (DIR), as well as data from Germany and Korea (see Table 5), the total DLP for both cervical spine and soft‐tissue neck scan types is quite similar (NDRL 470 mGy.cm and 450 mGy.cm respectively) but the CTDI_vol_ is higher for cervical spine than for soft‐tissue neck. The NDRLS data portal has been updated to ensure that data for these scan types will be collected separately into the future.

Chest–abdomen–pelvis scans are frequently performed as two separate scan series, an arterial chest phase followed by a portal venous abdomen phase. The NDRLs for this protocol assume the reporting of the total DLP and the average CTDI_vol_ across the component series. The NDRLs released in 2012 (Table 3) contained an anomaly in that the DLP (1200 mGy.cm) and CTDI_vol_ (30 mGy) implied a rather short scan length (40 cm) and the CTDI_vol_ was twice that for the individual chest and abdomen–pelvis scans (15 mGy). Figure 5 (B) shows the presence of these ‘short’ CAP scans. The presence of the peak at shorter apparent scan length (around 45 cm) in the distribution for dual‐series scans confirmed the suspicion that users were providing misleading data for CAP scans. After applying the relevant corrections, the new CTDI_vol_ NDRL for CAP is markedly lower (11 mGy) in comparison to the previous value (30 mGy) but similar to the new CTDI_vol_ NDRLs for chest (10 mGy) and abdomen–pelvis (13 mGy).

Provisional NDRLs have been established for kidney–ureter–bladder scans (non‐contrast, suspected renal colic) using the data collected for abdomen–pelvis scans. ARPANSA believes that the dose delivered during KUB scans is generally lower than abdominal‐pelvis scans and that, as a result, the provisional KUB values are conservative. A review of the KUB data will be undertaken in 2020 to establish updated NDRLs for this procedure that reflect actual observed practice.

As can be seen from Table 3, the new NDRLs for DLP are 12% to 26% lower than the previous NDRLs. Large reductions for CTDI_vol_ are seen in the case of CAP and soft‐tissue neck, as discussed above. Increasing use of iterative reconstruction technology has contributed to this reduction.

The ICRP and some national bodies have discussed using the 50^th^ percentile as an optimisation target, an ‘achievable dose’.^19^, ^24^, ^28^ The ICRP has also suggested that facilities with dose levels below the 25th percentile should pay particular attention to ensure that image quality is adequate.^19^ Table 3 shows the 25th and 50th percentiles of the FRL distributions for CTDI_vol_ and DLP along with the NDRLs (75th percentiles). The 25th and 50th percentiles have no official status in Australia; but are published on the ARPANSA website^22^ for information purposes to give facilities a better indication of how their dose levels compare.

Table 5 compares the new Australian adult MDCT NDRLs to those in other countries. The data suggest that Australian practice is generally consistent with comparable countries. In most cases the new Australian NDRLs are lower than those in the UK, USA and Japan, but a little higher than those in Germany. Dose levels for lumbar spine scans have reduced in comparison to the 2012 NDRLs but remain at the upper end in international comparisons.

The revised NDRLs provide updated benchmarks for facilities that are more consistent with current practice. National DRLs need to be updated routinely to ensure that they keep pace with changes in technology and practice as facilities apply the optimisation principle to maximise the benefit‐to‐risk ratio of their scans. ARPANSA expects to conduct another review of the data in 2021 to assess whether there have been further changes that would warrant an update of the NDRLs.

It is important to recognise that attention to dose for an imaging procedure is only one component of the optimisation process. It is vitally important that image quality is also assessed to ensure that it is sufficient for the diagnostic task. The NDRLs do not address this aspect. Facilities are encouraged to utilise the image quality self‐audit offered by the RANZCR to ensure that dose reduction is not achieved at the expense of diagnostic efficacy.^29^

## Conclusion

ARPANSA, in partnership with professional associations, has reviewed and revised the Australian NDRLs for multi‐detector CT for adult patients. The new NDRLs are lower than the previous NDRLs, and better reflect current practice among imaging facilities in Australia. The new Australian NDRLs are similar to those in other developed countries. It should be noted that due to the voluntary nature in submitting the surveys, the NDRLs could be biased. In addition, the KUB NDRLs are preliminary reference levels based on the abdomen–pelvis reference levels. ARPANSA will conduct a review of the KUB NDRLs in 2021. Use of the ARPANSA National Diagnostic Reference Level Service continues to increase, providing valuable feedback to imaging facilities on how dose levels for their scans compare to those of their peers.

## Funding

This work was supported by the Australian Government.

## Conflict of Interest

The authors declare that they have no conflict of interest.
